# Postoperative Pain After Robot-Assisted Laparoscopic Ventral Hernia Repair

**DOI:** 10.3389/fsurg.2021.724026

**Published:** 2021-10-28

**Authors:** Per Lindström, Göran Rietz, Åsa Hallqvist Everhov, Gabriel Sandblom

**Affiliations:** ^1^Department of Clinical Science and Education Södersjukhuset, Karolinska Institutet, Stockholm, Sweden; ^2^Department of Surgery, Södersjukhuset, Stockholm, Sweden

**Keywords:** robot-assisted laparoscopy, ventral hernia, pain, transabdominal preperitoneal repair, transabdominal retromuscular repair, intraperitoneal onlay mesh

## Abstract

**Background:** Robot-assisted ventral hernia repair, when performed correctly, may reduce the risk for pain and discomfort in the postoperative period thus enabling shorter hospital stay. The aim of the present study was to evaluate postoperative pain following robot-assisted laparoscopic repair. The approach was selected after an intraoperative decision to complete the repair as: (1). Transabdominal Preperitoneal Repair (TAPP); (2). Trans-Abdominal RetroMuscular (TARM) repair; or (3). Intraperitoneal Onlay Mesh (IPOM) repair depending on anatomical conditions.

**Methods:** Twenty ventral hernia repairs, 8 primary and 12 incisional, were included between 18th Dec 2017 and 11th Nov 2019. There were 8 women, mean age was 60.3 years, and mean diameter of the defect was 3.8 cm. The repairs were performed at Södersjukhuset (Southern General Hospital, Stockholm) using the Da Vinci Si Surgical System®. Sixteen repairs were completed with the TAPP technique, 2 with the TARM technique, and 2 as IPOM repair.

**Results:** Mean hospital stay was 1.05 days. No postoperative infection was seen, and no recurrence was seen at 1 year. At the 30-day follow-up, fifteen patients (75%) rated their pain as zero or pain that was easily ignored, according to the Ventral Hernia Pain Questionnaire. After 1 year no one had pain that was not easily ignored.

**Conclusion:** The present study shows that robot-assisted laparoscopic ventral hernia is feasible and safe. More randomized controlled trials are needed to show that the potential benefits in terms of shorter operation times, earlier discharge, and less postoperative pain motivate the extra costs associated with the robot technique.

## Introduction

Ventral hernia, primary as well as incisional, is associated with severe morbidity. Repair of ventral hernia ranges from minor procedures that can be undertaken as day surgery to extremely complex reconstructions. There are several surgical techniques for treating ventral hernia, but they are all associated with risks and may lead to long-term postoperative pain and disability. The open approach has been used for a long time but is associated with physiological stress, postoperative pain, and high wound infection and seroma rates (1).

The minimally invasive laparoscopic technique using intraperitoneal onlay mesh is well-established and associated with fewer wound infections, but is sometimes followed by severe postoperative pain. Leaving a mesh in the abdominal cavity, as with the IPOM technique, carries the risk of interaction with the organs of the abdominal cavity (1). Other approaches have been developed to place the mesh outside the abdominal cavity such as endoscopic Mini- or Less-Open Sublay repair (eMILOS), endoscopic Totally Extraperitoneal Approach (TEA), TransAbdominal PrePeritoneal repair (TAPP), and enhanced-view totally extraperitoneal repair (eTEP) (2–4). These approaches are technically more complicated, but postoperative pain may be less since tension in the abdominal wall along the hernia defect is lower. Furthermore, mesh-related complications are less.

Another way to lower the risk for long-term postoperative pain is the use of robot-assisted laparoscopy (5). Robotic assistance facilitates dissection and also enables preperitoneal and retromuscular hernioplasty. The TAPP technique (2) implies that the preperitoneal space is entered via the abdominal cavity and that the peritoneum is closed over the mesh after it has been put in place. Laparoscopic TransAbdominal Retromuscular (TARM) repair is performed through a longitudinal incision in the peritoneum and posterior rectus sheath, providing access to the retromuscular space which enables placement of the mesh in a sublay position (6).

The hypothesis of the present study was that robot-assisted ventral hernia repair, if performed correctly, causes little pain and discomfort in the postoperative period enabling earlier discharge from hospital. By placing the hernia mesh in a physiologically and anatomically advantageous position, rapid recovery, and reduced costs related to hospital stay and sick leave were predicted, the assumption being that the technique is safe and practical in routine clinical practice. To evaluate this hypothesis we carried out a case series of robot-assisted laparosopic hernia repairs with a standard follow-up programme.

## Materials and Methods

Surgery was performed at Södersjukhuset using the Da Vinci Si Surgical System® (Intuitive Surgical, Sunnyvale, CA, USA). The decision, which approach was to be used, was made intraoperatively. One 12 mm trocar for the camera and two 8 mm trocars were used. The defect was closed with intracorporeal Stratafix® sutures or v-loc® sutures, and a Medtronic Progrip® or Symbotex® mesh was used. The primary goal was to complete the procedure as a TAPP or TARM repair, but the surgeon was prepared to use the IPOM technique or open repair if deemed necessary.

All procedures began with introduction of the trocars through the left abdomen wall lateral to the semilunar line. Hernia content and abdominal adhesions were reduced before taking the decision on which approach was to be used. The aim was to complete the procedure with TAPP or TARM approach, i.e., to place the mesh outside the abdominal cavity. The decision on whether to complete the repair with a TAPP/TARM or IPOM technique was based on the integrity of the peritoneal layer, size and number of defects, coexisting diastasis recti, and the width of the rectus muscles. The IPOM technique was used when the preperitoneal and retromuscular route was found to be impossible due to the anatomy. The team was also prepared to convert to open repair should IPOM repair prove impossible.

When the TAPP technique was deemed feasible, the peritoneal layer was opened from the abdominal cavity on the ipsilateral side of the hernia and an appropriately sized pocket, including the reduced hernia sack, was created. The hernia defect was then closed and a Progrip® mesh was placed in the pocket with the hooks directed toward the abdominal wall. Finally the peritoneal incision was closed with a running suture.

When the TARM technique was chosen, the same principle and mesh were used but the ipsilateral incision in the abdominal wall also included the posterior rectus sheath. Care was taken to keep the peritoneal layer in the midline intact. The hernia sack was reduced and the posterior rectus sheath on the contralateral side was opened and dissection completed. When TAPP or TARM repair was performed, the hernia was reduced and a Progrip® mesh was placed into the accessible anatomical layer. When the mesh had been placed, the peritoneum was closed using continuous resorbable sutures.

If the peritoneal layer was torn while performing TAPP, or if TARM was not feasible but the abdominal contents of the hernia sac could be reduced, an IPOM repair was performed. When IPOM repair was performed, the hernia defect was closed and a Symbotex® mesh with an adhesion barrier was applied over the defect. The IPOM mesh was sutured to the abdominal wall with absorbable sutures using the double-crown technique. Regardless of which technique was used, the hernia defect was closed with running Stratafix® 0.

All patients were followed up at the outpatient clinic after 30 days and at 1 year. The patients were given a clinical examination and answered the Ventral Hernia Pain Questionnaire (VHPQ), a validated instrument intended for follow up of patients who undergo ventral hernia repair (7). Complications were graded according to Dindo et al. (8).

## Results

Between December 18th 2017 and November 12th 2019, twenty ventral hernia repairs, eight primary and 12 incisional, were included. Twelve men and eight women, mean age 60.3 years, standard deviation 10.0 years. Five patients were obese, two patients had diabetes mellitus, one patient had sclerodermia, one had hemiparesis following an intracranial bleeding andone had multiple sclersosis. One patient was classified as ASA I, 14 as ASA II and 5 as ASA III.

The mean size of the defect was 3.8 cm in diameter. The distribution of the hernias according to the European Hernia Society classification (9) is shown in Tables 1, 2.

**Table 1:** **(A)** Distribution of primary ventral hernias according to the EHS classification (9), **(B)** Distribution of incisional hernias according to the EHS classification (9).

	**Hernia defect diameter**		
**(A)**			
	**<2 cm**	**2–4 cm**	**>4 cm**
Umbilical	0	3	0
Epigastric^*^	0	1	4
**(B)**			
	**<4 cm**	**4–10 cm**	**>10 cm**
Midline incisional hernias	7	3	0
Lateral incisional hernias	1	1	0

* *Two patients had concomitant umbilical and epigastric hernias, they are included in the epigastric hernia group*.

**Table 2:** Ventral hernia pain questionnaire 30 days and 1 year postoperatively.

	**30 days postoperatively**	**One year postoperatively**
	***N* (%)**	***N* (%)**
Number of responders	20 (100%)	17 (85%)
**Level of pain when responding to the questionnaire**	13 (65%)	14 (70%)
No pain		
Pain present but easily ignored	2 (10%)	2 (10%)
Pain that could not be ignored, but did not interfere with daily activities	3 (15%)	0 (0%)
Pain that interfered with concentration on daily activities	1 (5%)	0 (0%)
Pain that interfered with most daily activities	1 (5%)	0 (0%)
Pain that necessitated bed rest	0 (0%)	0 (0%)
Pain that forced to seek immediate medical attention	0 (0%)	0 (0%)
No response	0 (0%)	4 (20%)
**Most intense pain perceived the last week**	13 (65%)	16 (80%)
No pain		
Pain present but easily ignored	2 (10%)	0 (0%)
Pain that could not be ignored, but did not interfere with daily activities	3 (15%)	0 (0%)
Pain that interfered with concentration on daily activities	1 (5%)	0 (0%)
Pain that interfered with most daily activities	1 (5%)	0 (0%)
Pain that necessitated bed rest	0 (0%)	0 (0%)
Pain that forced to seek immediate medical attention	0 (0%)	0 (0%)
No response	0 (0%)	4 (20%)
**Frequency of pain attacks last week**	4 (20%)	
A few times		
Several times		
Every day	2 (10%)	
Every day and night	1 (5%)	
Constant pain		
Not applicable (no pain reported)	13 (65%)	16 (80%)
No response	0 (0%)	4 (20%)
Duration of pain attacks		N/A
No pain	5 (25%)	
A few minutes	1 (5%)	
**Most of the day**	4 (20%)	
All day		
Constant pain		
Not applicable (no pain reported)	13 (65%)	16 (80%)
No response	0 (0%)	4 (20%)
**Which of the following activities have been affected by abdominal pain**	2 (10%)	0 (0%)
Rising from a low-sitting chair		
Sitting for more than 30 min	1 (5%)	0 (0%)
Standing for more than 30 min	0 (0%)	0 (0%)
Climbing stairs	1 (5%)	0 (0%)
Driving a car	1 (5%)	0 (0%)
Sport activities	2 (10%)	0 (0%)
No response		4 (20%)
**Pain medication for abdominal pain the last week**	2 (10%)	1 (5%)
Yes		
No	16 (80%)	11 (55%)
No response	2 (10%)	8 (40%)
**Sick-leave due to abdominal pain the last 2 months**	12 (60%)	11 (55%)
No		
Yes	4 (20%)	0 (0%)
No response	4 (20%)	9 (45%)
**Perception of stiffness or rigidity in the abdominal wall?**	15 (75%)	13 (65%)
No		
Yes	3 (15%)	3 (15%)
No response	2 (10%)	4 (20%)
**Satisfied with the operation**	0 (0%)	0 (0%)
No		
Yes	19 (95%)	15 (75%)
No response	1 (5%)	5 (25%)
**Prepared to undergo the procedure once more if necessary**	0 (0%)	0 (0%)
No		
Yes	19 (95%)	14 (70%)
No response	1 (5%)	6 (30%)

*All 20 patients responded to the Ventral Hernia Pain Questionnaire (VHPQ) 30 days after surgery and 17 (85%) at the 1-year follow-up*.

No conversion to open repair was necessary. Sixteen repairs were completed with the TAPP technique and two with the TARM technique. In two cases, the mesh could not be placed extraperitoneally, IPOM was therefore chosen. There was no conversion to open repair. In 19/20 (95%) cases, an overlap of at least 4 cm was achieved.

Mean hospital stay was 1.05 days (19 patients 1 day, 1 patient 2 days). No postoperative infection was seen. One patient had postoperative urinary retention requiring an indwelling catheter (Clavien-Dindo II). Eight patients (40%) developed a postoperative seroma that persisted at least 30 days and one patient had a seroma that lasted 1 year (Clavien-Dindo II). No recurrence was seen at the 1-year follow-up.

All 20 patients responded to the Ventral Hernia Pain Questionnaire (VHPQ) 30 days after surgery and 17 (85%) at the 1-year follow-up (Table 2). No recurrence was found at clinical investigation 1 year postoperative. No radiologic examinations were carried out to detect recurrences. After 30 days, fifteen patients (75%) had no pain or pain that was easily ignored. After 1 year no one hade pain that was not easily ignored.

## Discussion

The present study shows that robot-assisted laparoscopic ventral hernia is feasible and safe. Robot-assisted laparoscopy enables atraumatic dissection and accurate placement and fixation of the mesh. Hospital stay was short (mean 1.05 days). Most patients (65%) were pain-free and only 2 patients needed analgesics at the 30-day follow-up. The risk for long-term pain thus appears to be lower than after conventional laparoscopic repair. However, randomized controlled trials are required to confirm this.

The relative benefit of robot-assisted ventral hernia repair is probably more pronounced if the mesh is placed extraperitoneally such as the sublay position or preperiotoneally. With increasing awareness of the complexity of abdominal wall anatomy, these approaches may become first hand alternatives in the future. These techniques can also be achieved with conventional laparoscopy, but require greater technical skill. Furthermore, the relative benefit of robot-assisted laparoscopy would probably be more obvious in large and complex incisional hernia repairs, though confirmation of this in a controlled study must be difficult in this very heterogenous group.

The relatively low prevalence of persisting pain in this group may be explained by the more atraumatic dissection enabled by the robot. These results should, however, be interpreted with caution since there was no control group and most of the hernia defects were relatively small. Time to discharge was, however, reduced to less than half the period when conventional laparospoic hernia repair was routine. Before 2017, most patients stayed at least 2 days postoperatively (data not shown).

The present study was conducted at an early stage in the learning curve of the surgeons (GR and PL) performing the procedures. Initially TAPP was the ideal choice, whereas IPOM was considered a rescue procedure in the event of peritoneal tear. This explains why the majority of cases in this study were completed as TAPP procedures. Subsequently the surgeons became acquainted with the TARM technique, making three approaches possible. TAPP and TARM were considered methods of choice whenever feasible since these techniques enable placement of mesh outside the abdominal cavity. The main drawback of the TAPP technique is that the peritoneal layer varies in thickness and is sometimes very thin and fragile. Provided the layers do not tear, TAPP is suitable and less invasive than TARM. On the other hand, TARM is more robust than the TAPP technique. TARM may have other drawbacks; it is not suitable, for example, if a diastasis recti is wide, and after previous surgery the peritoneal layer in the midline may be very thin. The choice of technique also depends on the size of the defect and thereby the size of the mesh which must fit the pocket created.

The heterogeneity of the group is another limitation of the study. The study included patients with primary ventral hernias as well as incisional hernias. TAPP and TARM is usually easier to accomplish for primary ventral hernias, which makes comparisons between these groups biased.

Robot-assisted hernia repair in routine clinical practice is still a controversial issue. Randomized controlled trials are needed to show that the potential benefits, i.e., shorter operation time, earlier discharge, and less postoperative pain, motivate the costs associated with the robot device. A recently published randomized controlled trial showed that the outcome was equal after robot-assisted and conventional laparoscopic hernia repair, but to greater costs with robot-assisted repair (10). The trial was, however, based on intraperitoneal mesh in both groups, which limits the relative advantage of robot-assisted repair. Extraperitoneal placement of the mesh is more easily accomplished with robot-assisted technique than with conventional laparoscopic technique.

It may be anticipated that rapid development of software and equipment required for robot-assisted surgery will lead to a decrease in cost. According to the guidelines for laparoscopic treatment of ventral and incisional abdominal wall hernias released by the International Endohernia Society, robot-assisted laparoscopy could be an option for ventral hernia repair and coexisting diastasis. This technique enables wide extraperitoneal mesh augmentation, minimal fixation, myofascial release, posterior component separation, and complete fascial closure. This reduces recurrence rate and length of stay but increases the rate of seroma (11). The level of evidence for its use, however, is relatively low. The main issue is probably anatomical circumstances where robot assistance may provide the solution.

## Data Availability Statement

The raw data supporting the conclusions of this article will be made available by the authors, without undue reservation.

## Ethics Statement

The studies involving human participants were reviewed and approved by Stockholm Ethical Review Board. The patients/participants provided their written informed consent to participate in this study.

## Author Contributions

PL assembled the cohort together with GR, carried out the follow up and drafted the manuscript. GR assembled the cohort together with PL and participated in writing the manuscript draft. ÅE supervised the study, participated in designing it, and participated in writing the manuscript draft. GS designed the study together with ÅE, financed it and finalized the manuscript draft. All authors contributed to the article and approved the submitted version.

## Funding

The authors declare that this study received funding from Intuitive Surgical Inc. The funder was not involved in the study design, collection, analysis, interpretation of data, the writing of this article or the decision to submit it for publication.

## Conflict of Interest

The authors declare that the research was conducted in the absence of any commercial or financial relationships that could be construed as a potential conflict of interest.

## Publisher's Note

All claims expressed in this article are solely those of the authors and do not necessarily represent those of their affiliated organizations, or those of the publisher, the editors and the reviewers. Any product that may be evaluated in this article, or claim that may be made by its manufacturer, is not guaranteed or endorsed by the publisher.
